# Functional impairment and quality of life in newly diagnosed adults attending a tertiary ADHD clinic in Ireland

**DOI:** 10.1007/s11845-024-03713-6

**Published:** 2024-05-17

**Authors:** Dimitrios Adamis, Sam West, Jasmin Singh, Liadan Hanley, Iulian Coada, Geraldine McCarthy, Natasha Langan, Blánaid Gavin, Fiona McNicholas

**Affiliations:** 1 Sligo Mental Health Services, Clarion Rd, Sligo, Ireland; 2 University of Galway, Galway, Ireland; 3 University College Dublin, Dublin, Ireland; 4 University of Limerick, Limerick, Ireland; 5 University College Dublin, Lucena CAMHS Rathgar, CHI Crumlin Dublin 12, Dublin 7, Dublin, Ireland

**Keywords:** ADHD, Adult, Attention Deficit-Hyperactive Disorder, Functional impairment, Quality of life

## Abstract

**Background:**

Attention Deficit-Hyperactive Disorder (ADHD) is a neurodevelopmental disorder, often persisting into adulthood.

**Aims:**

To investigate the levels of functionality and quality of life (QoL) in adult patients newly diagnosed with ADHD and to compare with those without an ADHD diagnosis.

**Methods:**

Consecutive patients who were referred to and assessed in a tertiary adult ADHD clinic enrolled in the study. Diagnosis of ADHD was based on DSM-5 criteria. Functionality was measured using the Weiss Functional Impairment Rating Scale (WFIRS) and the Global Assessment of Functioning Scale (GAF). QoL was assessed with the Adult ADHD Quality of Life Questionnaire (AAQoL).

**Results:**

Three-hundred and forty participants were recruited, 177 (52.1%) females. Of them 293 (86.2%) were newly diagnosed with ADHD. Those with ADHD had significant lower functionality as it was measured with the WFIRS and GAF, and worse QoL (AAQoL) compared to those without. In addition, a significant correlation between GAF and WFIRS was found.

**Conclusions:**

The results show that adults with ADHD have decreased functionality and worse QoL when compared against those presenting with a similar symptomatology, but no ADHD diagnosis. ADHD is not just a behavioural disorder in childhood, but a lifelong condition with accumulating problems that can lead to lower QoL and impaired functioning throughout adulthood.

## Introduction

Attention Deficit-Hyperactivity Disorder (ADHD) is a neurodevelopmental disorder characterised by either significant symptoms of inattention or hyperactivity and impulsiveness or a combination of the two. The current diagnostic criteria for ADHD include six or more symptoms of inattention and six or more symptoms of hyperactivity–impulsivity, but for older adolescents and adults (from 17 years and above), five symptoms are required [1]. The pooled prevalence of ADHD in the general adult population is estimated at around 2.5% [2], but in selective populations like psychiatric outpatient clinics, the pooled prevalence is increased to 14.61% [3]. Although it has been seen as a childhood disorder which is believed to remit in adolescence, evidence is evolving of persistence into adult life [4]. Symptoms and associated functional impairments have been shown to fluctuate throughout adulthood in approximately 90% of cases [5]. Previous studies have shown that adults with ADHD have more functional impairment compared to healthy controls in several areas of functioning [6, 7]. This is not surprising as the DSM-5 criteria for ADHD, in addition to the presence of the core symptoms, requires (criterion D) that those symptoms interfere or reduce social, academic, or occupational functioning [1]. Therefore, level of functioning is an important aspect of the diagnosis of ADHD. Studies which have investigated and compared functionality of adults with ADHD to those with a mixture of other mental disorders showed that adults with ADHD had significantly worse functioning [7]. Moreover, it has been hypothesised that, in people with ADHD, this functional impairment may be more important in the causal pathway of stress, anxiety and mood than the specific symptoms of ADHD per-se [8].

Quality of Life (QoL) is a related construct to functional impairment which has long been explored in children with ADHD and is recently gained increasing attention in adults with ADHD. QoL refers to individuals’ subjective perceptions of their lives and is influenced by their culture and values [9]. The QoL typically focuses on and evaluates physical, psychological, social, and environmental factors. The presence of a disorder generally reduces the QoL, especially in cases of comorbidities or more severe disorder trajectories [10]. Previous longitudinal and cross-sectional studies have shown that the QoL of adults diagnosed with ADHD is lower compared to normal controls [10–12]. Treatment of ADHD improves both QoL and ADHD symptomatology [13–15].

Therefore, functional impairment and QoL are becoming increasingly recognized as important clinical components when considering how best to understand, support and treat adult ADHD. In this paper, we investigate functionality and QoL in a cohort of adults who have been referred to an ADHD clinic in Ireland for evaluation and treatment. The aims of the present study are: (a) to investigate and present the levels of functional impairment and QoL in adults newly diagnosed with ADHD, (b) to compare the levels of functionality and QoL between those diagnosed with ADHD and those who presented with similar symptomatology but do not fulfil the criteria for ADHD and (c) to investigate if there are differences in functionality and QoL among the different ADHD subtypes (combined, inattentive, hyperactive/impulsive). A secondary aim is to examine the concurrent validity (corelation) of the two function scales that we used in the present study: the Weiss Functional Impairment Rating Scale (WFIRS) and the Global Assessment of Functioning Scale (GAF).

## Methods

### Design

Cross-sectional, observational study.

### Setting

Consecutive outpatients attending an Adult ADHD clinic. The adult ADHD clinic is a tertiary level service. Referrals are accepted from the Adult Mental Health Services (AMHS) in line with the Republic of Ireland Health Service Executive (HSE) model of care [16]. These guidelines direct that patients are screened in AMHS using the Adult ADHD Self-Report Scale (ASRS) [17], and Wender Utah Rating Scale (WURS) [18] scales. Those screening positive on both scales were referred to the ADHD Adult Clinic for further clinical diagnostic assessment and treatment.

### Inclusion–exclusion criteria

All referred and consented patients were eligible for inclusion. Exclusion criteria were (a) patients with severe learning disability, or severe brain injury, and (b) patients not able to speak or read in the English language.

### Measurements/Scales

#### Demographics

Demographic data provided by the respondent, included age, gender, marital status, employment status, and higher educational achievement.

#### The diagnostic interview for adult ADHD (DIVA 5)

The DIVA 5 is a semi-structured instrument with good correlation with other self-reported rating scales and is well-validated. It is based on the DSM-V diagnostic criteria for ADHD and is designed to aid clinical diagnosis of ADHD in adults [19]. It takes about 60 to 90 min to be completed.

#### Psychiatric clinical evaluation

All participants also had a psychiatric clinical evaluation according to DSM-5 criteria. The psychiatrist used all available information (not blind to the administered scales, including DIVA 5), collateral history from parents or other family members (where possible) and detailed semi-structured neurodevelopmental history.

#### Weiss functional impairment rating scale (WFIRS)

WFIRS is a self-reported scale and consists of 69 items which covers seven domains of functioning (family, work, school, life skills, self-concept, social, and risks). There is four-point Likert rating scale for each item ranging from zero (never or not at all) to three (very often, very much). Mean scores can be calculated by omitting items with a missing or ‘not applicable’ response [20]. The total mean scores of WFIRS ranging from 0 to 3 and a higher total mean score (or on each domain/subscale) indicates greater functional impairment. The psychometrics for this scale are characterised as good and it has been widely used in research and clinical practice [21, 22].

#### Adult ADHD quality of life questionnaire (AAQoL)

AAQoL was developed and validated to measure quality of life in patients with ADHD [23]. The psychometrics of the scale have been investigated in different studies and it is considered as a valid and reliable instrument [23–25]. It consists of 29 items with each item rated by patients on a five-point Likert scale ranging from 1 to 5. It yields a total score (based on all items) and four subscale scores: life productivity, psychological health, life outlook, and relationships. After reversing scores and transforming them to a scale from 0 to 100, higher scores indicate better quality of life.

#### Global assessment of functioning (GAF)

The GAF was initially developed by Luborsky [26] as a Health Sickness Rating Scale and was subsequently modified and included as axis V in DSM-III–R and DSM-IV. It has been dropped from the DSM-5 but remains widely used due to its simplicity and good psychometrics [27]. GAF scores ≤ 70 indicate clinically significant functional impairment [26]. It is rated by the clinician for evaluating a person’s psychological, social, and occupational functioning on a hypothetical continuum of mental health problems ranging from 1, representing the hypothetically sickest individual, to 100, representing the hypothetically healthiest. The scale is divided into 10 equal parts and provides defining characteristics (both symptoms and social functioning) for each 10-point interval. It has been validated in different settings, and its reliability is estimated to be 0.78 [27–29].

### Ethics

The Local Research Ethics Committee approved the study. The procedures and rationale for the study were explained to all participants and written informed consent obtained.

### Statistical analyses

Statistical analysis was conducted using the IBM (SPSS) v25. For the first aim of the study, descriptive statistics were reported. Continuous variables are reported as means plus standard deviation, while categorical variables are reported as counts and percentages. If continuous variables were non-normally distributed, the median, minimum, maximum and Interquartile Rage (IQR) were reported. Comparison between the two groups (ADHD vs no-ADHD, second aim of the study) was conducted with a parametric (*t*-tests) for the normally distributed variables WFIRS and AAQoL and with a non-parametric test (Mann–Whitney test) for the non-normally distributed GAF. To compare the outcome variables functional impairment and quality of life among the three ADHD subtypes (third aim of the study), one way ANOVA for the normally distributed variables WFIRS and AAQoL, and the Kruskal–Wallis test for the non-normally distributed GAF were used. For the fourth aim of the study (concurrent validity of WFIRS and GAF), given that GAF was non-normally distributed, a Spearman’s rho test was performed. Finally, for the post hoc power calculations, the G*Power 3.1 program was used [30].

## Results

### Descriptive statistics

#### Demographics

The mean age of participants (*n* = 340) was 30.78 (SD: 10.475), median 29, Interquartile Range (IQR) 17, minimum 18, maximum 62 years old, with 177 (52.1%) females. The majority were single 207 (64.7%), 57 (18.0%) married, co-habiting 38 (11.9%), separated 15 (4.7%), divorced 3 (0.9%), and *n* = 20 did not give this information (missing data). Regarding the employment status, most of them were currently employed 138 (42.8%), unemployed 94 (29.6%), students 84 (26.4%), few retired 4 (1.3%) and 22 were missing data. The highest educational level achieved was, none 5 (1.6%), junior certificate 5 (1.8%) leaving certificate 160 (51.9%), vocational diploma 24 (7.8%), other diplomas 20 (6.5%), non-university degree (Institutes of Technology) 27 (8.8%), and university degree/postgraduate degree 67 (21.8%). Table 1 shows those demographics separately for those diagnosed with ADHD and those without an ADHD diagnosis, who did not.

**Table 1:** Marital status, employment, and education levels for those diagnosed with and without ADHD and results of *x*^2^ tests

		Diagnosis according to DSM-5				
		No (*n* = 47)		Yes (*n* = 293)		
		*n*	%	*n*	%	
Marital status	Single	31	66.0	176	64.5	*x*^2^ = 2.793 df: 4 *p* = .593
	Married	8	17.0	49	17.9	
	Co-habiting	4	8.5	34	12.5	
	Separated	4	8.5	11	4.0	
	Divorced	0	0.0	3	1.1	
Employment	Currently employed	22	48.9	114	41.8	*x*^2^ = 1.388 df: 3 *p* = .708
	Unemployed	11	24.4	83	30.4	
	Student	11	24.4	73	26.7	
	Retired	1	2.2	3	1.1	
Highest level of education	None	1	2.2	4	1.5	*x*^2^ = 3.250 df: 6 *p* = .777
	Junior certificate	0	0.0	5	1.9	
	Leaving certificate	25	54.3	135	51.5	
	Vocational diploma	5	10.9	19	7.3	
	Other diplomas	1	2.2	19	7.3	
	Non university degree	4	8.7	23	8.8	
	University/postgraduate degree	10	21.7	57	21.8	

#### ADHD diagnosis

Using the DIVA 5 and clinical psychiatric evaluation (based on DSM-5 criteria) 293 participants (86.2%) met criteria for ADHD diagnosis and 47 (13.8%) did not. The reasons for non-diagnosis were because either the number of symptoms was subthreshold, or the age of onset of symptoms was later than the 13 years old or the symptoms were better explained by another mental disorder. From those diagnosed with ADHD, the majority were diagnosed with combined subtype (*n* = 169, 57.7%), inattentive subtype (*n* = 117, 39.9%) and few with hyperactive/impulsive subtype (*n* = 7, 2.4%).

#### Functional impairment and quality of life (WFIRS, AAQoL, GAF)

The descriptive statistics of these three scales and their subscales are presented separately for those diagnosed with ADHD and those not diagnosed (Table 2). The median and the IQR are also given as the GAF was not normally distributed. It can be seen in Table 2 that people with ADHD have lower QoL from the midpoint of the scale (50). The functionality as measured by the WFIRS and GAF, was a little better than the midpoints of the scales (1.5 and 50 respectively), indicated substantial functional impairment overall.

**Table 2:** Descriptive statistics of the scales WFIRS, AAQoL, GAF and their subscales

		Diagnosis according to DSM-5			
		No (*n* = 47)		Yes (*n* = 293)	
		Mean	S D	Mean	S D
WFIRS	Family	.94	.630	1.20	.691
	Work	.99	.506	1.38	.716
	School	1.53	.764	1.96	.720
	Life skills	1.47	.640	1.71	.579
	Self-concept	1.94	.657	2.21	.785
	Social	1.03	.712	1.16	.718
	Risk	.64	.616	.61	.533
	Total WFIRS	1.13	.463	1.35	.479
AAQoL	Life productivity	44.91	21.099	33.60	18.550
	Psychological health	42.59	24.193	36.05	20.071
	Life outlook	48.96	16.274	41.36	16.228
	Relationships	58.06	23.018	47.33	20.506
	Total AAQoL	47.90	17.211	38.35	14.566
GAF	GAF	65.29 (median 65 IQR:15)	13.631	58.39 (median 60 IQR:15)	11.052

### Bivariate statistics

#### Correlation between GAF and WFIRS

GAF demonstrated a significant corelation with total WFIRS (Spearman’s *r* =  − 0.312, *p* < 0.001) as well as with its subscales (family, *r* =  − 0.224, *p* < 0.001; work *r* =  − 0.295, *p* < 0.001 school, *r* =  − 0.300, *p* < 0.001; life-skills *r* =  − 0.127, *p* = 0.043; self-concept, *r* =  − 0.140, *p* = 0.030: social, *r* =  − 0.256, *p* < 0.001; risk, *r* =  − 0.168, *p* = 0.008).

#### Comparison between those diagnosed with and without ADHD on demographic characteristics

No differences were found between those two groups regarding age (Mann–Whitney *U* = 6681.0, *z* =  − 0.327, *p* = 0.744), gender (*x*^2^ = 0.213, df:1, *p* = 0.644), marital status, employment status and level of education using *x*^2^ tests (see Table 1).

#### Comparison between those diagnosed with and without ADHD on each of the three scales (WFIRS, AAQoL, GAF)

Table 3 (*t*-tests) shows the results from those comparisons of the total scales and their subscales. Those diagnosed with ADHD had worse functional impairment (WFIRS) and worse quality of life compared to those without ADHD. Because GAF was not normally distributed the Mann–Whitney test was used for the comparison. Similarly, those with ADHD had significantly worse functioning as measured with the GAF compared to those without (Mann–Whitney *U* = 3562.5, z =  − 3.115, *p* = 0.002).

**Table 3:** *T*-test between those with and without ADHD in WFIRS and AAQoL and their subscales

	*t*	df	Sig	Mean difference	Std. error difference	95 C. I	
						Lower	Upper
Family	− 2.382	312	**.018**	− .2594	.1089	− .4737	− .0451
Work	− 4.052	68.219	**.000**	− .3823	.0943	− .5706	− .1940
School	− 2.953	208	**.004**	− .4227	.1431	− .7049	− .1404
Life skills	− 2.522	303	**.012**	− .2395	.0949	− .4263	− .0526
Self-concept	− 2.007	288	**.046**	− .2657	.1323	− .5262	− .0051
Social	− 1.113	288	.267	− .1373	.1234	− .3803	.1056
Risk	.301	291	.763	.0279	.0926	− .1544	.2103
Total WFIRS	− 2.898	312	**.004**	− .2206	.0761	− .3704	− .0708
Life productivity	2.471	258	**.014**	11.3063	4.5755	2.296	20.316
Psychological health	1.315	258	.190	6.5440	4.9761	− 3.255	16.343
Life outlook	1.911	19.622	.071	7.5992	3.9762	− .7053	15.903
Relationships	2.123	258	**.035**	10.7277	5.0525	.7783	20.677
Total AAQoL	2.651	258	**.009**	9.5579	3.6047	2.4594	16.656

#### Functional impairment and quality of life among the ADHD subtypes

Finally, ANOVA tests with Bonferroni corrections were applied for the normally distributed variables (WFIRS and AAQoL) and Kruskal–Wallis test for GAF to find out if there were differences in the functional impairment and quality of life among the subtypes. The results for WFIRS and AAQoL are presented in Table 4. Those with combined subtype have significantly worse functioning compared to those with hyperactive/impulsive or inattentive subtype but no significant difference between hyperactive/impulsive and inattentive subtype. Similarly, those with combined subtype have worse quality of life compared to hyperactive/impulsive but no difference compared to inattentive subtype. Regarding the GAF the Kruskal–Wallis test did not show any significant difference among the groups (Kruskal–Wallis H = 2.343, df:2, *p* = 0.340).

**Table 4:** ANOVA test multiple comparisons with Bonferroni corrections

Dependent variable	(I) ADHD subtypes	(J) ADHD subtypes	Mean difference (I-J)	Std. error	Sig	95 C. I	
						Lower bound	Upper bound
WFIRS	Combined	Hyperactive/Impulsive	.486	.1932	**.038**	.0202	.951
		Inattentive	.229	.0582	**< .001**	.0891	.369
	Hyperactive/Impulsive	Inattentive	− .256	.1947	.567	− .7256	.212
AAQoL	Combined	Hyperactive/Impulsive	− 18.486	6.513	.**015**	− 34.19	− 2.78
		Inattentive	− 3.572	1.897	.183	− 8.147	1.01
	Hyperactive/Impulsive	Inattentive	14.914	6.568	.072	− .922	30.75

### Post hocpower calculations

For the comparisons between those diagnosed with and without ADHD (*t*-tests) the outcome variable WFIRS had a power of 0.833 to detect true difference the AAQoL a power of 0.686 and for the GAF the observed power was 0.90.

For the last analysis (ANOVA tests), a power calculation was performed for each of the two variables (WFIRS and AAQoL). For the WFIRS, the power to detect true differences was 0.981 and for AAQoL 0.830. For the comparison between the groups Combined subtype vs. Hyperactive/Impulsive for the WFIRS, the power was 0.965 and for the AAQoL was 0.841.

## Discussion

The results above show that adults newly diagnosed with ADHD have low QoL and substantial functional impairment as measured by the AAQoL and WFIRS. The impairments in the QoL of individuals with ADHD as well as of their family (parents) are a frequent finding in previous studies which have mainly focused on children and adolescents with ADHD. A meta-analysis of 7 studies in people with ADHD showed greater impairment in QoL compared to those with a normal development [31] and another meta-analysis [32] of 17 studies showed that the parents of children with ADHD had reduced QoL. To date, there is no specific meta-analysis for adults, however, the few studies which investigate QoL in adults with ADHD reported similar results with the present study [11, 12]. It was suggested that the QoL in adults with ADHD is related to developmental impairment in executive function (EF) [33] while impaired EF has also been demonstrated to predict depression [34], a common comorbidity in adults with ADHD. In a more recent study [35] showed that ADHD could affect QoL in a cumulative manner indirectly via EF in addition to the negative impact of depressive/anxiety symptoms on QoL. It is reasonable to hypothesise that the long-standing symptomatology of ADHD combined with varied combinations of comorbidities suboptimal coping mechanisms, repeated experience of challenge and failure in the everyday life, underachievement, may contribute in a cumulative fashion to low QoL. It is worth noting that in this study the participants’ AAQoL total mean score (38.35) was lower than reported in other studies [23, 25, 33]. However, it is reasonable to assert that the participants in our study have long standing disorder unmedicated and untreated and perhaps with more severe consequences given that the vast majority were diagnosed for the first time in their adulthood, (the time that they participated in the study), and thus had not previously accessed ADHD treatment. In addition, given that this patient cohort was referred from general adult mental health teams, they may have had more comorbidities relative to cohorts in previous studies.

Similarly, the functional impairment (WFIRS) in the investigated sample was relatively lower than the midpoint, but a range of other functional outcomes was also impaired (low employment rate, low educational level, majority single) illuminating the sequelae of unrecognised and untreated ADHD in relation to work, educational attainment and relationships. These findings are in accordance with previous studies [11, 36, 37]. A number of studies which examined the EF of adults with ADHD proposed that impairment of EF partly explain the educational and occupational problems [7, 38], but some other studies contradict this suggestion [11]. However, ADHD is characterised by heterogeneous cognitive profiles, which can and do change across time [39, 40]. Therefore, the suggestion of a direct or indirect link between EF and functional impairment is rather premature and needs to be investigated further particularly with longitudinal studies.

The results of this study also demonstrate that adults with ADHD with similar sociodemographic variable as those who have symptoms of ADHD but do not meet the criteria for diagnosis, have lower levels of QoL and functionality. A previous study by Pawaskar [41] reported contradictory findings with the present study; however, it is worth noting that there were major methodological differences between the two studies. Pawaskar et al.’s study, comprised a bigger sample but was based mainly on self-reported diagnoses with no clinical evaluation performed. In our study, both groups were comprehensively clinically evaluated. If our results are replicated in further studies, it may indicate that the symptoms of ADHD in and of themselves are not the only explanation for the low QoL and impaired functioning evident in adult ADHD. Rather, it is reasonable to infer that the early onset ADHD during critical developmental phases in childhood adversely affects developmental trajectories including suboptimal coping, social, and educational outcomes, thereby lowering functional capacity and worsening QoL in the adulthood [42].

In addition, our results indicate that there are differences in the levels of QoL and functionality among the ADHD subtypes. Those with combined subtype have significantly worse functioning compared to those with hyperactive/impulsive and to those with inattentive subtype but this significance remains only for the hyperactive/impulsive when the QoL examined. Only a small number of studies in adults have specifically examined the ADHD-subtypes in relation to functional impairment and QoL. An early study[43], reported similar results to ours, regarding functional impairment and QoL, with two more studies examining functional impairment alone reporting similar results [44, 45]. In children and adolescents, there are more studies which have explored functional impairment and ADHD subtype, with similar results reported [46, 47]. Given that the combined subtype includes both symptom clusters relating to inattention and hyperactivity it may be reasonable to predict that functionality and QoL would be more negatively impacted. It is worth highlighting that the number of hyperactive/impulsive subtype is small (*n* = 7, 2.4%); however, this is in keeping with expected rate in adulthood and even in childhood the rate of hyperactive/impulsive subtype is low, estimated at 6 to 8% of the total ADHD population [43]. Given that hyperactivity is reduced with age, it is likely that the prevalence of hyperactive/impulsive subtype in our sample is representative and with adequate power to detect true difference.

It is worth also to comment here about the gender distribution. ADHD is considered mainly as a disorder frequent in males than females especially in childhood and adolescent, with a reported male to female ratio of 3:1 to 9:1 [48]. More recent studies narrow this gap [49]. However, in adult ADHD the rate of male female is higher for females. Previous studies in adult referred populations for ADHD shows similar rates with the present study [3, 50]. Among the explanations that have been suggested for this reverse rate in adulthood are that in childhood the over representation of males may reflect under diagnosis in females, which somewhat correct itself, with the likelihood of females being more likely than males to seek out treatment as an adult (referral bias), genetic vulnerability, endocrine factors, psychosocial contributors, increased levels of remission of symptoms of ADHD in males, or late onset of ADHD more prominent in females [51, 52].

Finally, our results demonstrate a significant corelation between WFIRS and GAF although this corelation is weak (31%). A higher corelation (moderate) was reported from a previous study in which WFIRS, translated to Japanese, was evaluated [53]. The concurrent validity of those two scales was not among our primary aims for the present study, but searching the literature it was found that GAF has been used in adult ADHD as an objective measurement of functioning by clinicians. WFIRS is designed specifically for populations with ADHD, and it is a self-reported scale. This weak correlation can be explained from the different construct that those two scales used and measured. GAF is more restrictive to psychological, occupational, and social impairment, while WFIRS is broader and examined seven domains of functioning. Besides the WFIRS is a self-reported scale and self-biases are possible [54].

### Limitations of the study

Although the sample was adequate with high observed power the comparison group of participants with ADHD-like symptoms was relatively small to allow firm conclusions especially for the AAQL scale (observed power 68.6%). Similarly, the sample of hyperactive/impulsive subtype was small as discussed above; however, as noted, the rate was similar to what it was expected in an adult population and thus likely to be representative and with a good power to detect true difference. A final limitation is the generalizability of the results. The sample was from an Irish special adult ADHD clinic in which there are certain guidelines for referrals. Therefore, the results can apply in similar settings but perhaps not in other settings which use different systems of referrals.

In summary, in this study we found that adults with ADHD have impaired functionality and low quality of life. Those with combined type ADHD have more functional impairment compared to the other two subtypes and worse QoL compared to hyperactive/impulsive subtype. Low QoL and functional impairment in those with ADHD are significantly worse compared to those with ADHD-like symptoms but no diagnosis of ADHD. This reinforces the view that ADHD is not just a behavioural disorder in childhood, but a lifelong condition with accumulating problems and symptoms that can lead to persistent difficulties throughout adulthood.

## Data Availability

The data that support the findings of this study are available from the corresponding author, DA, upon reasonable request.
